# TIMP-1 in the prognosis of patients who underwent coronary artery bypass surgery: a 12-year follow-up study

**DOI:** 10.3389/fcvm.2023.1226449

**Published:** 2023-12-14

**Authors:** Jui-Tzu Huang, Shih-Hsien Sung, Chiao-Po Hsu, Chern-En Chiang, Wen-Chung Yu, Hao-Min Cheng, Cheng-Hsiung Huang

**Affiliations:** ^1^ Division of Cardiology, Department of Medicine, Taipei Veterans General Hospital, Taipei, Taiwan; ^2^ Institute of Emergency and Critical Care Medicine, National Yang Ming Chiao Tung University College of Medicine, Taipei, Taiwan; ^3^ Cardiovascular Research Center, National Yang Ming Chiao Tung University College of Medicine, Taipei, Taiwan; ^4^ Department of Internal Medicine, National Yang Ming Chiao Tung University College of Medicine, Taipei, Taiwan; ^5^ Division of Cardiovascular Surgery, Department of Surgery, Taipei Veterans General Hospital, Taipei, Taiwan; ^6^ General Clinical Research Center, Taipei Veterans General Hospital, Taipei, Taiwan; ^7^ Center for Evidence-Based Medicine, Taipei Veterans General Hospital, Taipei, Taiwan; ^8^ Department of Medical Education, Taipei Veterans General Hospital, Taipei, Taiwan

**Keywords:** TIMP-1, MMP, coronary artery disease, subclinical inflammation, ventricular remodeling

## Abstract

**Introduction:**

Matrix metalloproteinases (MMPs) and tissue inhibitors of metalloproteinases (TIMPs) have been linked to clinical outcomes in patients with coronary artery disease (CAD). However, the prognostic value of TIMP-1 in patients with CAD who underwent coronary artery bypass grafting (CABG) has not been elucidated. We aimed to investigate the correlations of TIMP-1 with high-sensitivity C-reactive protein (hs-CRP) and N-terminal pro-brain natriuretic peptide (NT-proBNP) in the long-term prognosis of consecutive patients who underwent CABG.

**Methods:**

A total of 234 patients (age: 70.4 ± 10.5 years, 84.6% men) with CAD who underwent CABG were prospectively enrolled. Preoperative levels of MMPs, TIMP-1, hs-CRP, and NT-proBNP were recorded. Major adverse cardiovascular events (MACE) were defined as non-fatal myocardial infarction, non-fatal stroke, and cardiovascular death.

**Results:**

During a median follow-up of 12.1 years, 120 deaths were recorded. The deceased were older, had more manifest acute coronary syndrome (ACS), a lower left ventricular ejection fraction (LVEF), and an estimated glomerular filtration rate (eGFR), but significantly higher MMP13, TIMP-1, hs-CRP, and NT-proBNP compared with the survivors. After adjusting for age, sex, manifest ACS, eGFR, LVEF, total cholesterol, and triglycerides, TIMP-1 (hazard ratio and 95% confidence intervals per SD: 1.506, 1.183–1.917), hs-CRP (1.349, 1.183–1.561), and NT-ProBNP (1.707, 1.326–2.199) were all independently associated with all-cause mortality. The mediation analysis revealed that the mortality risks of TIMP-1 were partially mediated by NT-proBNP (62.2%) and hs-CRP (25.3%). The associations of TIMP-1 with MACE were partially mediated by NT-proBNP (54.4%) but not hs-CRP.

**Conclusions:**

TIMP-1 was an independent predictor of long-term outcomes after CABG, with possible roles in subclinical inflammation and postoperative cardiac remodeling.

## 1. Introduction

Matrix metalloproteinases (MMPs) are involved in the degradation of the extracellular matrix (ECM), which participates in various biological processes such as angiogenesis, inflammation, and tissue remodeling (1, 2). The tissue inhibitors of metalloproteinases (TIMPs) can modulate the local activities of MMPs. An imbalance in the expression of MMPs and TIMPs is associated with atherosclerotic cardiovascular disease (1–3). Epidemiological studies have demonstrated that the serum levels of MMPs and TIMPs, along with their ratio, are associated with cardiovascular events and long-term survival in individuals with or without coronary artery disease (CAD) (4–6). In addition, both MMPs and TIMPs have been correlated with longitudinal left ventricular remodeling in patients with hypertension (7) and in the general population (8), which is linked to incident heart failure (HF) (9). Although Morishita et al. presented the prognostic value of circulating MMPs and TIMPs in patients with HF (10), it is hypothesized that the clinical benefits observed with sacubitril/valsartan in the PARADIGM-HF trial were due to its regulation of the extracellular matrix (11). Cardiac remodeling has been associated with long-term survival in patients with severe CAD who have undergone coronary artery bypass surgery (CABG) (12, 13). However, the clinical value of circulating MMPs and TIMPs in predicting adverse outcomes remains unclear. Subclinical inflammation, which affects endothelial function and microvascular blood flow, may increase the risk of cardiovascular complications and mortality after CABG (14, 15). Therefore, the present study aimed to investigate the prognostic value of MMPs and TIMP and their association with inflammation and HF in patients who underwent CABG. We investigated the correlation of TIMP-1 with high-sensitivity C-reactive protein (hs-CRP) and N-terminal pro-brain natriuretic peptide (NT-proBNP) levels to predict long-term outcomes in consecutive patients who underwent CABG.

## 2. Materials and methods

### 2.1. Study population

A total of 346 patients referred for CABG by the same experienced surgeon between January 2001 and December 2007 were eligible for this study. Medical history, anthropometric measurements, coronary artery angiography findings, and fasting blood samples were obtained preoperatively. Patients who experienced acute myocardial infarction or unstable angina within 30 days prior to CABG were defined as having acute coronary syndrome (ACS). Moreover, 87 patients with ongoing infectious diseases, concomitant surgical procedures other than CABG, or active malignancy were excluded from the study. Furthermore, 25 patients were lost to follow-up. Finally, 234 patients were included in this analysis. This was performed following the relevant guidelines and regulations. The study conformed to the principles outlined in the Declaration of Helsinki, and the study protocol was approved by the Institutional Review Board of the Taipei Veterans General Hospital. Informed consent was obtained from all participants.

### 2.2. Assessments of biochemistry and biomarkers

Overnight fasting serum and plasma samples were obtained on the day of surgery for measurement of lipid profiles, renal function, and biomarkers. The estimated glomerular filtration rate (eGFR) was calculated by a modified Modification of Diet in Renal Disease equation based on a Chinese population using plasma creatinine (PCr, mg/dl) as eGFR (ml/min/1.73 m^2^) = 175 × PCr ^−1.234^ × age ^−0.179^ × (0.79 if the subject was a woman) (16). NT-proBNP (Biomedica, Vienna, Austria), hs-CRP (Dade Behring, Marburg, Germany), MMPs, and TIMP-1 (R&D Systems, Abingdon, United Kingdom) levels were determined using commercially available enzyme-linked immunosorbent assays.

### 2.3. Clinical outcomes

The study population was followed up through outpatient clinics, telephone contacts, reviews of medical records, and the National Death Registry to identify clinical events. Patients who died within 30 days of surgery were defined as having surgery-related mortality and were categorized as having cardiovascular death. Major adverse cardiovascular events (MACEs) were defined as non-fatal myocardial infarction, non-fatal stroke, and cardiovascular death.

### 2.4. Statistical analysis

Data from this study were expressed as mean ± standard deviation for continuous variables and as percentages for categorical variables. Comparisons between groups were performed using the Student's *t*-test for continuous variables and the chi-square test for categorical variables. Associations between different biomarkers were evaluated by calculating Pearson correlation coefficients. The determinants of TIMP-1 were evaluated using the forward stepwise selection of multiple linear regression. Additionally, the relative contribution of different markers was calculated as the percentage of the individual partial R^2^ divided by the model R^2^. Survival curves were plotted using the Kaplan–Meier method and assessed using overall and pairwise log-rank tests. Predictors of all-cause mortality were determined using Cox proportional hazards regression analysis, and a multivariable stepwise model was constructed with entry criteria of *P* < 0.5 and *P* < 0.15 to stay in the model. Hazard ratios (HR) and 95% confidence intervals (CI) per standard deviation were presented for each biomarker. The biomarker cutoff points for the prediction of mortality were defined using receiver operating characteristic (ROC) curve analyses. NT-proBNP was logarithmically transformed before linear regression analysis due to the skewed distribution. Mediation analysis was used to assess the importance of pathways and direct and indirect causal effects between different biomarkers and outcomes using the R package (17–21). All analyses were conducted by SAS 9.4 (IBM, Cary, NA, USA) and RStudio Team (2021) (PBC, Boston, MA, USA). The figures were plotted using MedCalc for Windows, version 19.4 (MedCalc Software, Oostende, Belgium). Differences were considered statistically significant at a two-tailed *P* < 0.05.

## 3. Results

The baseline characteristics of the study population are presented in Table 1. Of 234 study participants (70.4 ± 10.5 years, 84.62% men, and 42.74% with diabetes), 108 experienced a MACE during a median follow-up duration of 12.1 years. In addition, a total of 120 deaths occurred, 92 of which were from cardiovascular causes. The deceased patients were characterized by older age, more ACS, a lower body mass index, left ventricular ejection fraction (LVEF), and eGFR compared to survivors. The distributions of male subjects, hypertension, diabetes, and active smokers, along with lipid profiles, were similar between the two groups. Patients with more A-grafts and complete revascularization during surgery had better survival rates. Moreover, the deceased group had significantly higher levels of hs-CRP, NT-proBNP, MMP-13, and TIMP-1 than the survivors. The serum levels of MMP-2 and MMP-9 were comparable.

**Table 1:** Baseline characteristics of the study population.

Variable	All (*N* = 234)	Dead (*N* = 120)	Alive (*N* = 114)	*P* value
Age, years	70.36 ± 10.47	73.26 ± 9.85	67.29 ± 10.26	<0.001
Men, %	198 (84.62)	99 (82.50)	99 (86.84)	0.357
Manifest ACS, %	82 (35.04)	55 (45.83)	27 (23.68)	<0.001
Hypertension, %	181 (77.35)	97 (80.83)	84 (73.68)	0.192
Diabetes mellitus, %	100 (42.74)	57 (47.50)	43 (37.72)	0.131
Smoker, %	82 (35.04)	40 (33.33)	42 (36.84)	0.574
Body mass index, kg/m^2^	24.68 ± 3.27	24.17 ± 3.15	25.21 ± 3.33	0.015
LVEF	0.47 ± 0.13	0.45 ± 0.14	0.51 ± 0.11	0.001
Characteristics of CABG				
Left main coronary artery disease %	66 (28.21)	39 (32.33)	27 (23.89)	0.157
Stenotic vessels	2.74 ± 0.52	2.74 ± 0.53	2.73 ± 0.52	0.988
Number of A-grafts	1.08 ± 0.34	1.02 ± 0.27	1.13 ± 0.39	0.016
Complete revascularization, %	189 (80.77)	84 (69.42)	105 (92.92)	<0.001
Preoperative biomarkers				
hs-CRP, mg/dl	0.90 ± 1.80	1.36 ± 2.33	0.42 ± 0.76	<0.001
NT-proBNP, fmol/ml	1,103 ± 1,222	1,559 ± 1,518	630.5 ± 473.9	<0.001
Preoperative biochemistry				
eGFR, ml/min/1.73 m^2^	62.70 ± 27.00	56.56 ± 28.16	69.16 ± 24.20	<0.001
Total cholesterol, mg/dl	178.1 ± 41.97	176.0 ± 46.82	180.2 ± 36.25	0.444
HDL-cholesterol, mg/dl	4.71 ± 2.08	4.95 ± 2.66	4.46 ± 1.23	0.08
LDL-cholesterol, mg/dl	1.77 ± 1.80	1.89 ± 2.53	1.64 ± 0.33	0.299
Triglycerides, mg/dl	146.6 ± 96.07	147.3 ± 103.8	145.9 ± 87.62	0.909
MMPs and TIMPs				
MMP-2, ng/ml	1.10 ± 2.28	1.35 ± 3.08	0.84 ± 0.76	0.11
MMP-9, ng/ml	62.99 ± 78.86	57.09 ± 73.72	69.14 ± 83.84	0.294
MMP-13, ng/ml	2.09 ± 1.24	2.26 ± 1.36	1.91 ± 1.09	0.036
TIMP-1, ng/ml	127.9 ± 63.67	141.0 ± 78.84	114.3 ± 38.53	0.002

ACS, acute coronary syndrome; eGFR, estimated glomerular filtration rate; HDL, high-density lipoprotein; hs-CRP, high-sensitivity C-reactive protein; LDL, low-density lipoprotein; LVEF, left ventricular ejection fraction; MMP, matrix metalloproteinases; NT-proBNP, N-terminal pro-brain natriuretic peptide; TIMP, tissue inhibitor of metalloproteinases.

### 3.1. Determinants of TIMP-1

**Figure 1:**
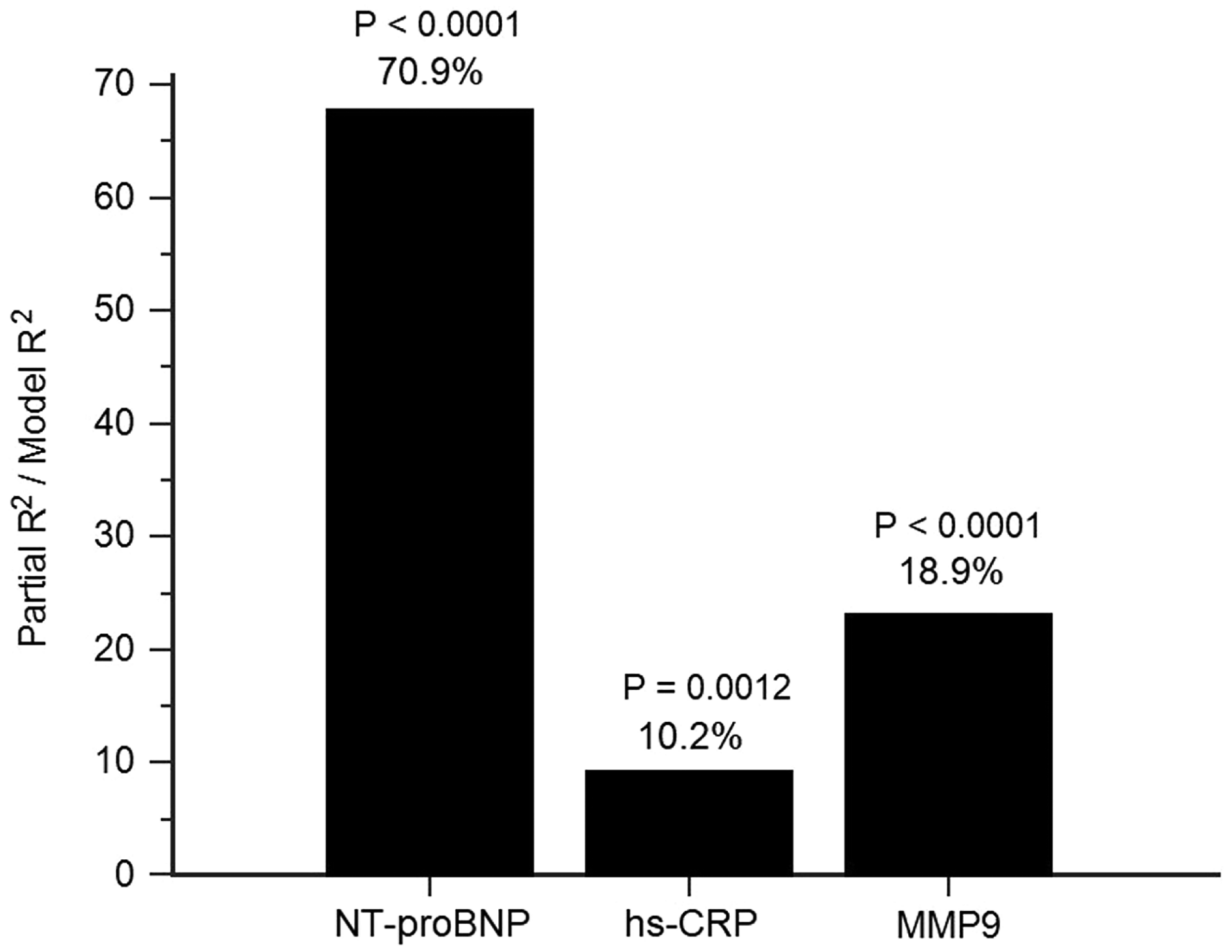
Determinants of tissue inhibitor of metalloproteinases (TIMP)-1. The attributed proportion of N-terminal pro-brain natriuretic pepetide (NT-proBNP), high-sensitivity C-reactive protein (hs-CRP) and matrix metalloproteinase (MMP)-9 to TIMP-1 was calculated by R^2^ of each biomarker over R^2^ of the forward selection regression model with fixed adjustments for age, sex, left ventricular ejection fraction, estimated glomerular filtration rate, acute coronary syndrome, total cholesterol and triglycerides.

With fixed adjustments for age, sex, LVEF, eGFR, ACS, total cholesterol, and triglycerides, using forward stepwise linear regression analysis, hs-CRP, NT-proBNP, and MMP-9 were all significant determinants of TIMP-1. Additionally, NT-proBNP had the largest partial R^2^, contributing 70.9% of the total variance in TIMP-1 (*P* < 0.0001), MMP-9 (18.9%, *P* < 0.0001), and hs-CRP (10.2%, *P* = 0.0012) levels (Figure 1).

### 3.2. Prognostic value of biomarkers

**Figure 2:**
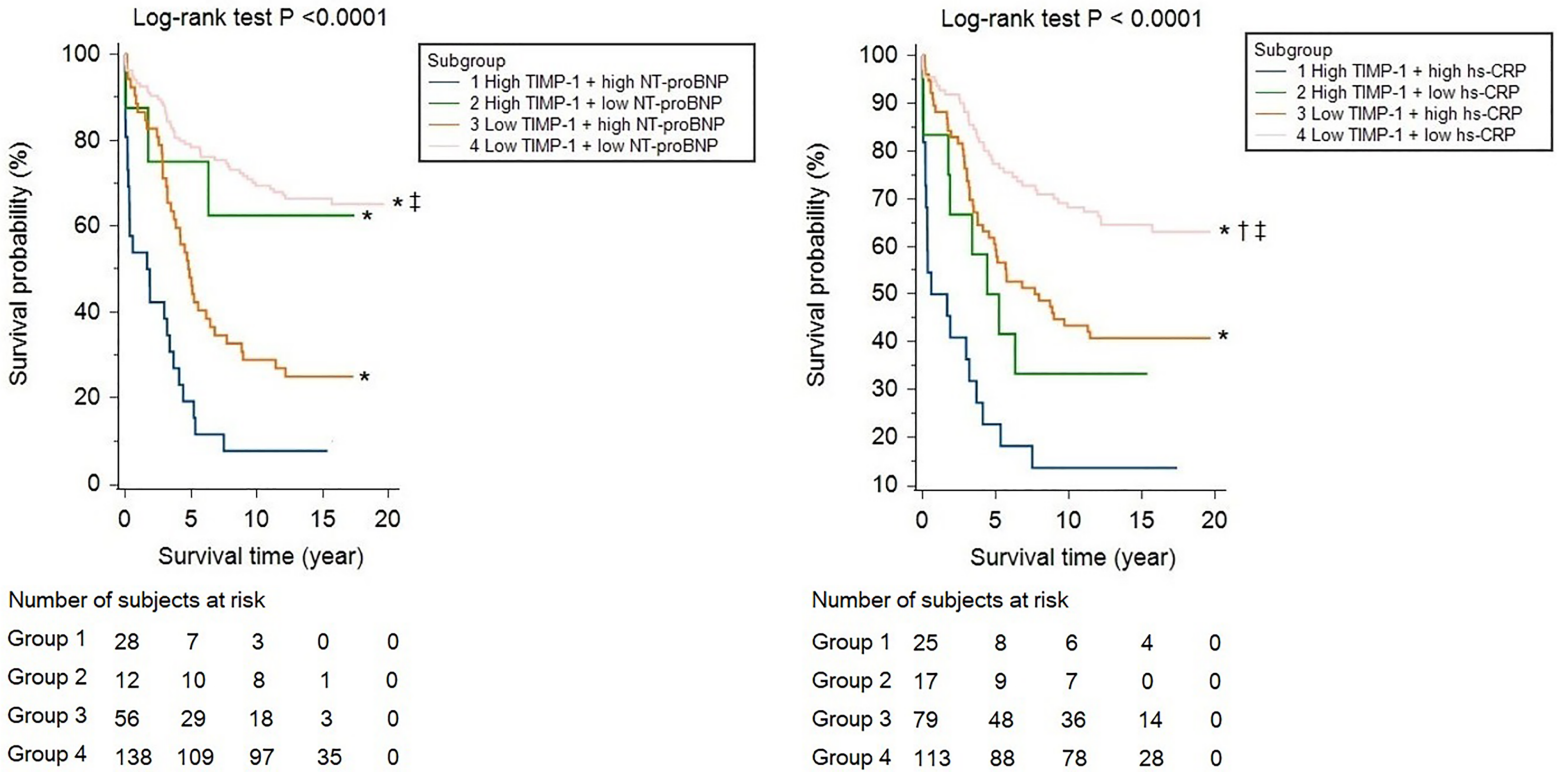
Kaplan-Meier survival curve analyses, stratified by high and low TIMP-1 (cut-off value: 128.4 ng/ml), in cooperation with NT-proBNP (cut-off value: 1548.8 fmol/ml) or hs-CRP (cut-off value: 0.57 mg/dl). * indicated a *P* value of <0.05 by pairwise log-rank test, compared with group 1, † for group 2, and ‡ for group 3.

Although age, ACS, body mass index, LVEF, and complete revascularization were associated with mortality, hs-CRP, and NT-proBNP levels were also crudely correlated with long-term survival (HR and 95% CI per-1SD: 1.409, 1.241–1.599 and 2.173, 1.787–2.642, respectively). TIMP-1 (1.525, 1.325–1.756) was also associated with all-cause mortality in the study population. (Supplementary Table S1). After adjusting for age and sex, hs-CRP, NT-proBNP, and TIMP-1 were independently associated with all-cause mortality and MACE (Tables 2, 3, Model 1). None of the MMP-2, MMP-9, or MMP-13 levels were independently linked to all-cause mortality or MACE during the follow-up period. After further adjustment for ACS, eGFR, LVEF, lipid profile, or complete revascularization, hs-CRP, NT-proBNP, and TIMP-1 remained significantly correlated with mortality and MACE. (Tables 2, 3, Model 2 and Model 3) By ROC curve analysis, the cut-off values of hs-CRP, NT-proBNP, and TIMP-1 for the prediction of mortality were 0.57 mg/dl, 1,548.8 fmol/ml, and 128.4 ng/ml, respectively. Patients with high TIMP-1 levels had significantly higher mortality rates after CABG than compared with the others (Supplementary Figure 1). Both high NT-proBNP and high hs-CRP would further deteriorate the long-term survival of patients with high TIMP-1 (Figure 2).

**Table 2:** Predictors of all-cause mortality in multivariable Cox regression analyses.

Variable	Univariate Model	Model 1	Model 2	Model 3
	HR (95% CI)	HR (95% CI)	HR (95% CI)	HR (95% CI)
MMP-2, 1SD = 2.3 ng/ml	**1.158** **(1.009–1.329)**	1.103 (0.947–1.285)	1.073 (0.915–1.259)	1.08 (0.925–1.26)
MMP-9, 1SD = 78.9 ng/ml	0.905 (0.726–1.126)	1.015 (0.81–1.273)	1.048 (0.84–1.309)	1.087 (0.872–1.355)
MMP-13, 1SD = 1.2 ng/ml	1.166 (0.973–1.396)	1.117 (0.935–1.336)	1.139 (0.94–1.381)	1.136 (0.943–1.369)
**TIMP-1**, 1SD = 63.7 ng/ml	**1.527** (**1.326–1.757)**	**1.605** (**1.386–1.858)**	**1.506** (**1.183–1.917)**	**1.546** (**1.222–1.957)**
**hs-CRP**, 1SD = 1.8 mg/ml	**1.409** (**1.242–1.600)**	**1.405** (**1.238–1.595)**	**1.349** (**1.165–1.561)**	**1.364** (**1.182–1.574)**
**^a^NT-proBNP**, 1SD = 0.37 fmol/ml	**2.174** (**1.788–2.644)**	**2.093** (**1.706–2.568)**	**1.707** (**1.326–2.199)**	**1.681** (**1.305–2.167)**

The bold values in the tables were emphasized to be statistically significant.

^a^NT-proBNP was log transformed.

Model 1: adjusted for age and sex.

Model 2: adjusted for age, sex, manifest acute coronary syndrome, estimated glomerular filtration rate, left ventricular ejection fraction, total cholesterol, and triglycerides.

Model 3: adjusted for age, sex, manifest acute coronary syndrome, estimated glomerular filtration rate, left ventricular ejection fraction, completed revascularization or not.

hs-CRP, high-sensitivity C-reactive protein; MMP, matrix metalloproteinases; NT-proBNP, N-terminal pro-brain natriuretic peptide; TIMP, tissue inhibitor of metalloproteinases. CI, confidence intervals; HR, hazard ratio; SD, standard deviation.

**Table 3:** Predictors of major adverse cardiovascular events in multivariable Cox regression analyses.

Variable	Univariate Model	Model 1	Model 2	Model 3
	HR (95% CI)	HR (95% CI)	HR (95% CI)	HR (95% CI)
MMP-2, 1SD = 2.3 ng/ml	1.099 (0.912–1.325)	0.993 (0.812–1.215)	0.959 (0.776–1.186)	0.992 (0.806–1.22)
MMP-9, 1SD = 78.9 ng/ml	0.946 (0.753–1.188)	0.999 (0.79–1.264)	1.024 (0.807–1.299)	1.127 (0.889–1.427)
MMP-13, 1SD = 1.2 ng/ml	1.057 (0.865–1.292)	1.027 (0.844–1.25)	1.008 (0.816–1.246)	1.019 (0.826–1.258)
**TIMP-1**, 1SD = 63.7 ng/ml	**1.552** (**1.347–1.788)**	**1.546** (**1.322–1.809)**	**1.41** (**1.093–1.819)**	**1.666** (**1.296–2.142)**
**hs-CRP**, 1SD = 1.8 mg/ml	**1.402** (**1.223–1.608)**	**1.387** (**1.212–1.587)**	**1.34** (**1.147–1.565)**	**1.398** (**1.194–1.638)**
**^a^NT-proBNP**, 1SD = 0.37 fmol/ml	**2.162** (**1.754–2.665)**	**1.792** (**1.449–2.215)**	**1.505** (**1.161–1.95)**	**1.699** (**1.296–2.227)**

The bold values in the tables were emphasized to be statistically significant.

^a^NT-proBNP was log transformed.

Model 1: adjusted for age and sex.

Model 2: adjusted for age, sex, manifest acute coronary syndrome, estimated glomerular filtration rate, left ventricular ejection fraction, total cholesterol, and triglycerides.

Model 3: adjusted for age, sex, manifest acute coronary syndrome, estimated glomerular filtration rate, left ventricular ejection fraction, completed revascularization or not.

hs-CRP, high-sensitivity C-reactive protein; MMP, matrix metalloproteinases; NT-proBNP, N-terminal pro-brain natriuretic peptide; TIMP, tissue inhibitor of metalloproteinases. CI, confidence intervals; HR, hazard ratio; SD, standard deviation.

### 3.3. Mediation analysis between biomarkers

**Figure 3:**
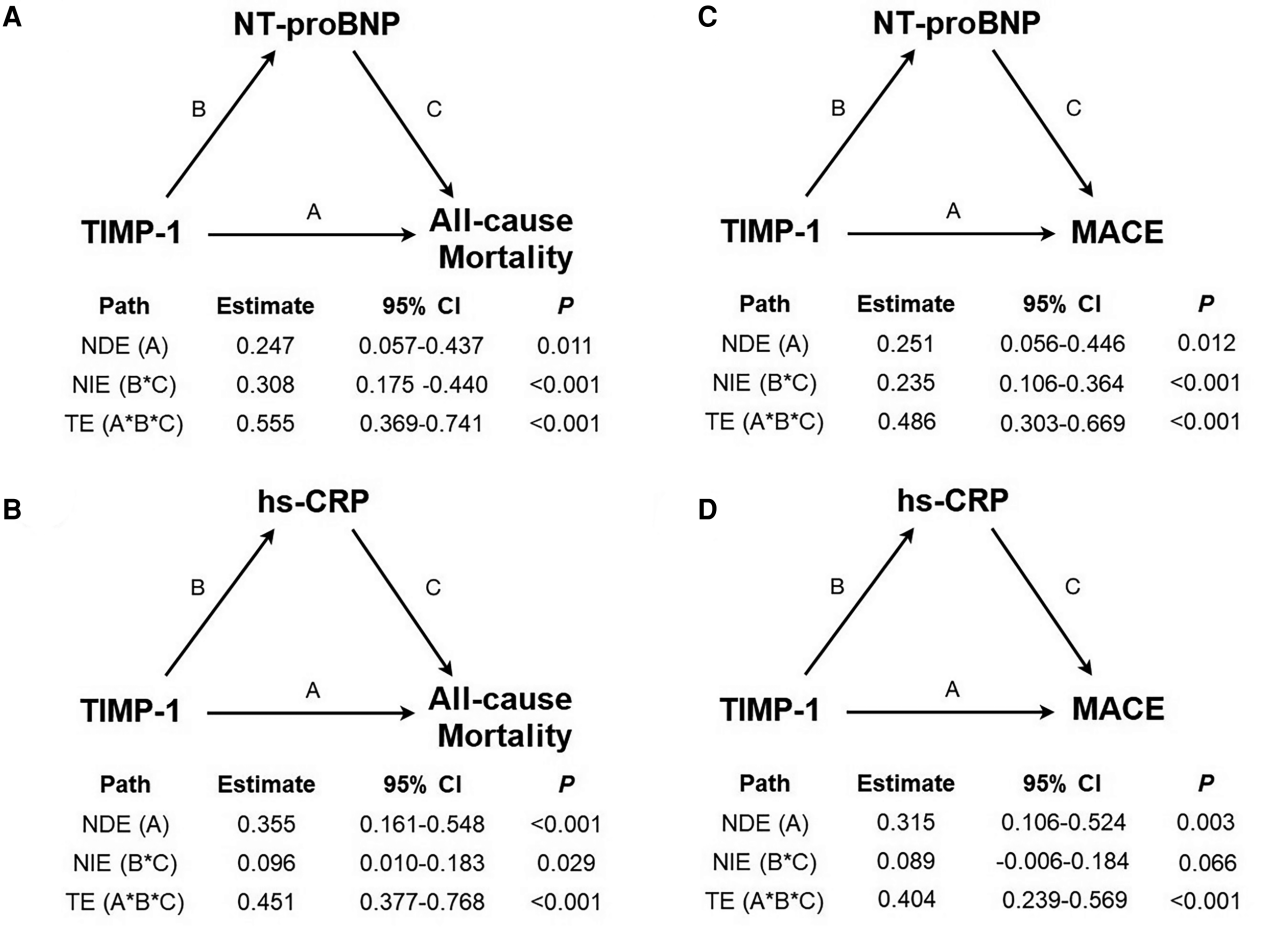
Causal-mediation analysis of tissue inhibitor of metalloproteinases (TIMP)-1, N-terminal pro-brain natriuretic pepetide (NT-proBNP) and high-sensitivity C-reactive protein (hs-CRP) in the prediction of mortality and major adverse cardiovascular events (MACE) after CABG. Effect estimates, 95% confidence intervals and P values were reported for all paths. (A: natural direct effect, B*C natural indirect effect, A*B*C total effect). (**A**) Mediator as NT-proBNP and outcome as all-cause mortality, proportion mediated = 62.2%. (**B**) Mediator as hs-CRP and outcome as all-cause mortality, proportion mediated = 25.3%. (**C**) Mediator as NT-proBNP and outcome as major adverse cardiovascular events , proportion mediated = 54.4%. (**D**) Mediator as hs-CRP and outcome as major adverse cardiovascular events , proportion mediated = 25.6%

Single-moderator mediation analysis using linear and Cox regression methods is presented in Figure 3, which shows a significant total effect of serum TIMP-1 level on all-cause mortality and MACE. The association between mortality and TIMP-1 level was mediated by NT-proBNP (62.2%) and hs-CRP (25.3%). In contrast, both NT-proBNP and hs-CRP levels were directly associated with an increased mortality risk. In addition, the association between TIMP-1 and MACE was mediated by NT-proBNP (54.4%) but not by hs-CRP. However, both NT-proBNP and hs-CRP levels were still directly associated with MACE.

## 4. Discussion

The present study demonstrated that TIMP-1, but not MMPs, is associated with MACE and long-term survival in patients with CAD who underwent CABG. In addition, both NT-proBNP and hs-CRP levels were correlated with clinical outcomes after CABG. Although both NT-proBNP and hs-CRP were related to serum levels of TIMP-1, causal mediation analysis demonstrated that NT-proBNP and hs-CRP may mediate the mortality risk of TIMP-1 on long-term outcomes in the study population.

### 4.1 Prognostic value of TIMP-1 after CABG

Although MMPs have been implicated in atherosclerotic plaque rupture, numerous studies have demonstrated that serum levels of MMPs correlate with clinical outcomes in patients with CAD (22–24). Furthermore, TIMPs, which counteract MMP activity, have also been associated with adverse cardiovascular events and the long-term survival of patients with CAD (4–6). In addition to plaque stability, both MMPs and TIMPs are expressed in cardiomyocytes and fibroblasts and are involved in cardiac remodeling (25–27). Patients with hypertension, left ventricular hypertrophy, or impaired left ventricular systolic function had higher serum TIMP-1 levels compared to their healthy counterparts (8, 28, 29). Lieb et al. further demonstrated that TIMP-1 was associated with incident HF in 922 Framingham participants during a 20-year follow-up period (9). Among patients with CAD who underwent CABG, the recurrence of atherosclerotic events and the development of HF may jeopardize long-term outcomes (30). Although subclinical inflammation may worsen microvascular function and contribute to graft failure and recurrent coronary events (15), remodeling of the left ventricle and/or concomitant HF exacerbate adverse events after CABG (31). The present study demonstrated that both hs-CRP and NT-proBNP are independent predictors of MACE and all-cause mortality in the study population. In addition, this study may be the first to demonstrate that TIMP-1 correlates with long-term clinical outcomes in patients who underwent CABG for more than 10 years. Although the prognostic value of MMPs has been validated in numerous populations with CAD (22–24), clinical outcomes related to MMPs after CABG have rarely been discussed. In a retrospective study of 200 patients who underwent CABG, Perek et al. demonstrated that tissue expression of MMP-2 in the saphenous veins was related to venous graft failure (32). No association between MMPs and long-term clinical outcomes has been previously reported. In the present study, no significant association between serum MMP levels and clinical outcomes after CABG was found. The reasons for this may include different MMP molecular constituents in the internal mammary artery and saphenous vein (33), and cardiopulmonary bypass may cause a transient increase in the concentration and activity of plasma MMPs (34).

### 4.2. TIMP-1, subclinical inflammation, cardiac remodeling, and outcomes

Hoseini et al. demonstrated a significant correlation between hs-CRP and TIMPs in patients with metabolic syndrome and atherosclerotic and cardiovascular diseases. Therefore, supporting the involvement of TIMPs in subclinical inflammation is an established risk factor for plaque instability and future coronary events (35). Opstad et al. demonstrated that the quartiles of serum TIMP-1 levels after myocardial infarction in 243 patients contributed to cardiac remodeling, including a large infarct size, high NT-proBNP levels, and poor LVEF obtained at 3 months (36) Nordeng et al. reported that TIMP-1 was highly expressed in intracoronary thrombi from 33 patients with ST-segment elevation myocardial infarction, mainly connected to monocytes and macrophages. Furthermore, both TIMP-1 in thrombi and leukocytes significantly correlated with peak troponin T levels, indicating its important role in early myocardial damage, remodeling, and inflammatory processes (37). Although the present study also showed that both hs-CRP and NT-proBNP were independently associated with TIMP-1, we further propose that NT-proBNP outweighs hs-CRP as a major determinant of TIMP-1. In addition, the prognostic value of serum TIMP-1 levels for long-term survival and MACE could be attributed to NT-proBNP and hs-CRP levels. The clinical associations of TIMP-1 were largely mediated by NT-proBNP. However, the study results suggest that TIMP-1 modulates ventricular remodeling and/or HF to impact long-term outcomes.

### 4.3. Potential limitations of the present study

This study had several limitations. First, the study population comprised Taiwanese people of Chinese descent, and the generalizability of the study results to non-Asians warrants further validation. Moreover, repeated measurements of biomarkers and myocardial function are lacking. Further studies are needed to determine how TIMP-1, in cooperation with subclinical inflammation or ventricular remodeling, exacerbates the clinical outcomes in patients with CAD who underwent CABG. Lastly, tissue expression of MMPs and TIMP-1 was not available in this study, and whether serum levels could reflect true biological activity is unknown.

## Data Availability

The raw data supporting the conclusions of this article will be made available by the authors, without undue reservation.
