# Supplementary material for: TIMP-1 in the prognosis of patients who underwent coronary artery bypass surgery: a 12-year follow-up study
Source: Front Cardiovasc Med. 2023 Dec 14;10:1226449. doi: 10.3389/fcvm.2023.1226449 (PMC10757603; doi:10.3389/fcvm.2023.1226449)
Supplement: Supplementary file 1 [file Table1.docx]

Supplementary Figure S1. Kaplan-Meier survival curve analyses of the study population, stratified by high and low tissue inhibitor of metalloproteinases-1 (TIMP-1, A), N-terminal pro-brain natriuretic peptide (NT-proBNP, B), and high sensitivity C-reactive protein (hs-CRP, C)


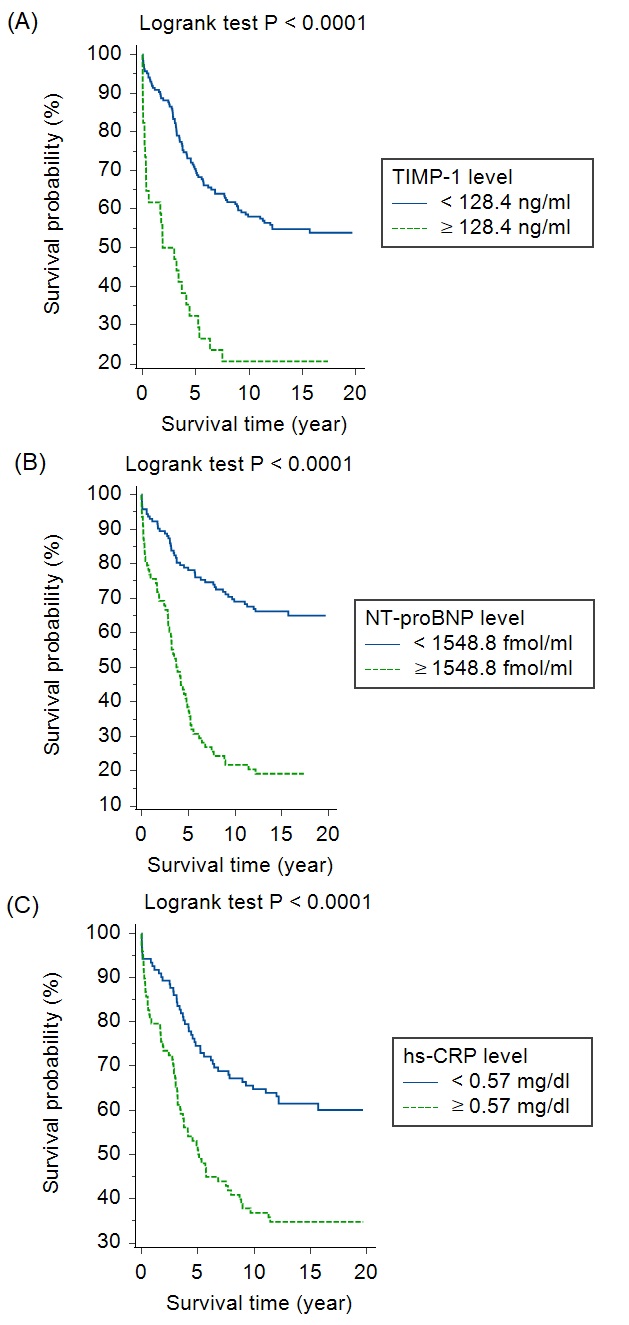


| Supplementary Table S1. Predictors of all-cause mortality and major adverse cardiovascular events (MACE) in the univariate Cox regression analyses | | |
| --- | --- | --- |
| Variable | All-cause mortality  HR (95% CI) | MACE  HR (95% CI) |
| Age, 1SD=10.5 years | **1.647 (1.301-2.084)** | **1.57 (1.232-2.0)** |
| Sex | 0.751 (0.469-1.203) | 0.582 (0.364-0.931) |
| LVEF, 1SD=0.1 | **0.643 (0.525-0.787)** | **0.75 (0.606-0.928)** |
| eGFR, 1SD=27.0 ml/min/1.73m^2^ | **0.654 (0.535-0.798)** | **0.619 (0.502-0.764)** |
| Manifest ACS | **2.16 (1.508-3.094)** | **1.723 (1.169-2.54)** |
| Triglycerides, 1SD=96.1 mg/dl | 0.981 (0.817-1.178) | 0.953 (0.78-1.165) |
| Total cholesterol, 1SD=42.0 mg/dl | 0.912 (0.744-1.119) | 0.993 (0.801-1.23) |
| Left main disease, % | 1.372 (0.936-2.009) | **1.732 (1.17-2.564)** |
| Number of A-grafts | **0.56 (0.326-0.961)** | 0.59 (0.335-1.041) |
| Complete revascularization, % | **0.352 (0.238-0.519)** | **0.335 (0.222-0.508)** |
| MMP-2, 1SD=2.3 ng/ml | **1.158 (1.009-1.329)** | 1.085 (0.906-1.299) |
| MMP-9, 1SD=78.9 ng/ml | 0.901 (0.724-1.122) | 0.894 (0.71-1.126) |
| MMP-13, 1SD=1.2 ng/ml | 1.169 (0.976-1.4) | 1.073 (0.881-1.307) |
| **TIMP-1**, 1SD=63.7 ng/ml | **1.525 (1.325-1.756)** | **1.495 (1.29-1.733)** |
| **hs-CRP**, 1SD=1.8 mg/ml | **1.409 (1.241-1.599)** | **1.376 (1.203-1.574)** |
| ***NT-proBNP**, 1SD=0.37 fmol/dl | **2.173 (1.787-2.642)** | **1.909 (1.561-2.335)** |
| *NT-proBNP was taken logarithm transformation  TIMP-1: metallopeptidase inhibitor 1, NT-proBNP: N-terminal pro-brain natriuretic peptide, hs-CRP: high sensitivity C-reactive protein | | |
